# MicroRNA-491 regulates the proliferation and apoptosis of CD8^+^ T cells

**DOI:** 10.1038/srep30923

**Published:** 2016-08-03

**Authors:** Ting Yu, Qian-Fei Zuo, Li Gong, Li-Na Wang, Quan-Ming Zou, Bin Xiao

**Affiliations:** 1 National Engineering Research Center of Immunological Products & Department of Microbiology and Biochemical Pharmacy, College of Pharmacy, Third Military Medical University, Chongqing 400038, China

## Abstract

T lymphocyte-mediated immune responses are critical for antitumour immunity; however, T cell function is impaired in the tumour environment. MicroRNAs are involved in regulation of the immune system. While little is known about the function of intrinsic microRNAs in CD8^+^ T cells in the tumour microenvironment. Here, we found that miR-491 was upregulated in CD8^+^ T cells from mice with colorectal cancer. Retroviral overexpression of miR-491 in CD8^+^ and CD4^+^ T cells inhibited cell proliferation and promoted cell apoptosis and decreased the production of interferon-γ in CD8^+^ T cells. We found that miR-491 directly targeted cyclin-dependent kinase 4, the transcription factor T cell factor 1 and the anti-apoptotic protein B-cell lymphoma 2-like 1 in CD8^+^ T cells. Furthermore, tumour-derived TGF-β induced miR-491 expression in CD8^+^ T cells. Taken together, our results suggest that miR-491 can act as a negative regulator of T lymphocytes, especially CD8^+^ T cells, in the tumour environment; thus, this study provides a novel insight on dysfunctional CD8^+^ T cells during tumourigenesis and cancer progression. In conclusion, miR-491 may be a new target for antitumour immunotherapy.

Immune responses are essential to protect against cancer. T lymphocytes, especially CD8^+^ cytotoxic T lymphocytes (CTLs), are key players in the restriction and elimination of tumour cells and tumour stromal cells^1^. A high density of CTLs in tumour tissue is usually beneficial for patients and correlates with patient outcome^2^,^3^,^4^,^5^. However, tumours have developed multiple strategies to thwart the antitumour immune response, such as the impairment of antigen presentation and processing machinery, the activation of negative costimulatory signals, and the promotion of antigen-specific T cell tolerance or dysfunction^6^. Tumour-infiltrating lymphocytes often exhibit an exhaustion profile. For example, effector CD8^+^ T cells cannot produce effector cytokines, such as interferon-γ (IFN-γ)^5^, or express specific inhibitory receptors, such as cytotoxic T lymphocyte-associated antigen (CTLA-4), programmed cell death 1 (PD-1) and T cell immunoglobulin- and mucin domain-containing molecule 3 (Tim-3)^7^,^8^. Thus, tumour-associated CD8^+^ T cells cannot effectively promote tumour rejection. However, the precise molecular mechanisms underlying T cell dysfunction during tumourigenesis and cancer progression are still poorly understood.

MicroRNAs (miRNAs) are small noncoding RNAs that play pivotal roles in the post-transcriptional regulation of genes during various biological processes, including immune cell development, homeostasis and responses^9^,^10^,^11^. Accumulating evidence suggests that miRNAs are intimately involved in the immunoregulation of antitumour responses. For example, TGF-β can induce the accumulation of chemokine (C-C motif) ligand 22 via the inhibition of miR-34a in the tumour environment, which results in the recruitment of regulatory T cells to suppress the immune response and contribute to immune escape^12^. In addition, miR-155 has been reported to act as a tumour suppressor by promoting CTLs accumulation and increasing IFN-γ production to limit tumour growth^13^,^14^. miR-19b and miR-17 are positive regulators of Th1 cell-mediated tumour rejection. They promote the proliferation of effector T cells, the production of IFN-γ, and the protection of cells from activation-induced cell death (AICD)^15^. These observations indicate that miRNAs are novel regulators of antitumour immunity and could be potential targets in cancer immunotherapy.

In the present study, we showed that miR-491 was one of the most highly upregulated miRNAs in splenic CD8^+^ T cells from colorectal tumour-bearing mice compared with their non-malignant counterparts. miR-491 has been reported to act as a tumour suppressor in various types of cancer^16^,^17^,^18^,^19^,^20^, but its function in the immune system is still unknown. Our data indicated that the overexpression of miR-491 could inhibit T cell proliferation, promote apoptosis and inhibit the production of IFN-γ in CD8^+^ T cells. In addition, we identified cyclin-dependent kinase 4 (CDK4), T cell factor 1 (TCF-1), and B-cell lymphoma 2-like 1 (Bcl2l1/Bcl-xL) as targets of miR-491 in CD8^+^ T cells. Furthermore, we discovered that miR-491 overexpression was induced by tumour-derived TGF-β. These results suggest that miR-491 can serve as a novel regulator of T cell function and that manipulation of miR-491 in CD8^+^ T cells will likely contribute to antitumour immunity.

## Results

### miR-491 expression was upregulated in CD8^+^ T cells from colorectal tumour-bearing mice

**Figure 1:**
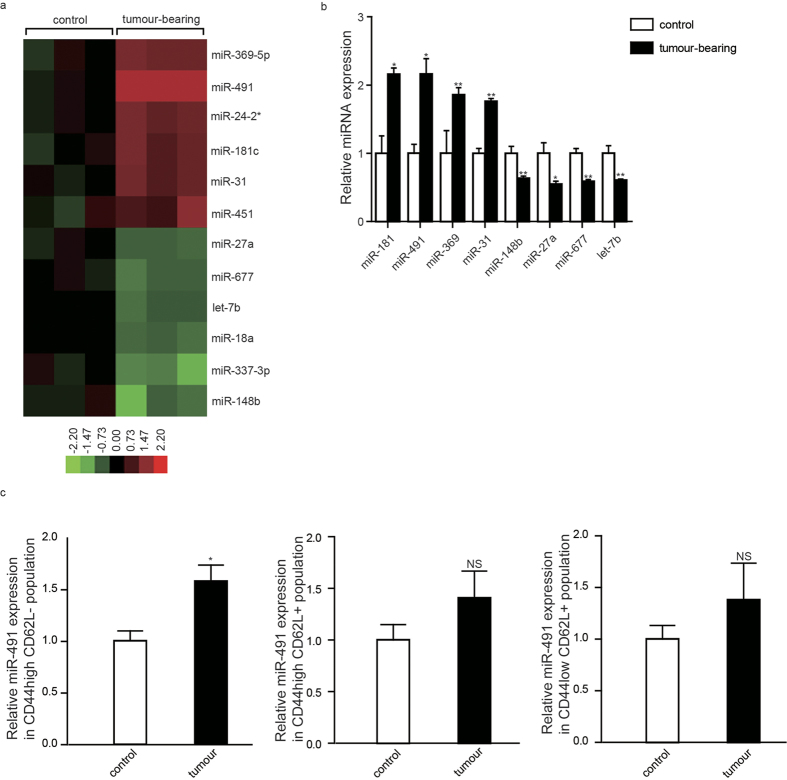
Differential expression of miRNAs in CD8^+^ T cells between colorectal tumour-bearing mice and controls. (**a**) Total RNA was extracted from splenic CD8^+^ T cell, and the miRNA profile analysis was performed with a high-throughput miRNA qPCR array. The heatmap shows the differential expression patterns of miRNAs in CD8^+^ T cells. **(b)** The expression levels of several selected miRNAs were validated by qRT-PCR in a repeated experiment. Small RNA U6 was used as an internal reference. (n = 5 *vs*. 5) **(c)** CD8^+^ T cell subsets from tumour-bearing mice and controls were sorted by FACS and expression of miR-491 in effector-like, memory and naïve CD8^+^ T cells between the two groups were detected with qPCR (n = 5 *vs*. 5). The data are represented as the mean ± SD. *P < 0.05; **P<0.01.

To investigate the effect of the tumour environment on the expression pattern of miRNAs in the immune system, we conducted a real-time PCR-based high-throughput miRNA array to identify a panel of differentially expressed miRNAs in total CD8^+^ T cells. Several miRNAs in splenic CD8^+^ T cells from colorectal tumour-bearing mice were significantly altered compared with their non-malignant counterparts, such as miR-369, miR-491, miR-181c, and miR-31 (Fig. 1a). miR-491 showed the highest upregulation by 2.2-fold than others (Fig. 1b). To investigate the expression abundance of miR-491 in CD8^+^ T cells, we detected miR-491 level compared with several miRNAs which had been reported to be functional in CD8^+^ T cells. The results showed that miR-491 steadily existed in CD8^+^ T cells but was not one of the most highly existed miRNAs (Fig. S1). To identify the original source of miR-491 upregulation, we further analysed miR-491 level in CD8^+^ T cell subsets between the two groups. Percentages of CD8^+^ T cell subsets are similar between tumour-bearing mice and controls (Fig. S2). As shown in Fig. 1c, miR-491 was upregulated in effector-like cells (CD44^high^ CD62L^−^) from tumour-bearing mice than controls (*P* < 0.05). And miR-491 had the tendency for increase in naive cells (CD44^low^ CD62L^+^) and central memory cells (CD44^high^ CD62L^+^) from tumour-bearing mice than controls but the differences did not reach statistical significance. These results indicated that miR-491 may be functional in effector like CD8^+^ T cells in tumour microenvironment. The role of miR-491 in CD8^+^ T cells during the antitumour immune response is still unknown. Therefore, we selected miR-491 for further investigation.

### miR-491 overexpression inhibited T cell proliferation

**Figure 2:**
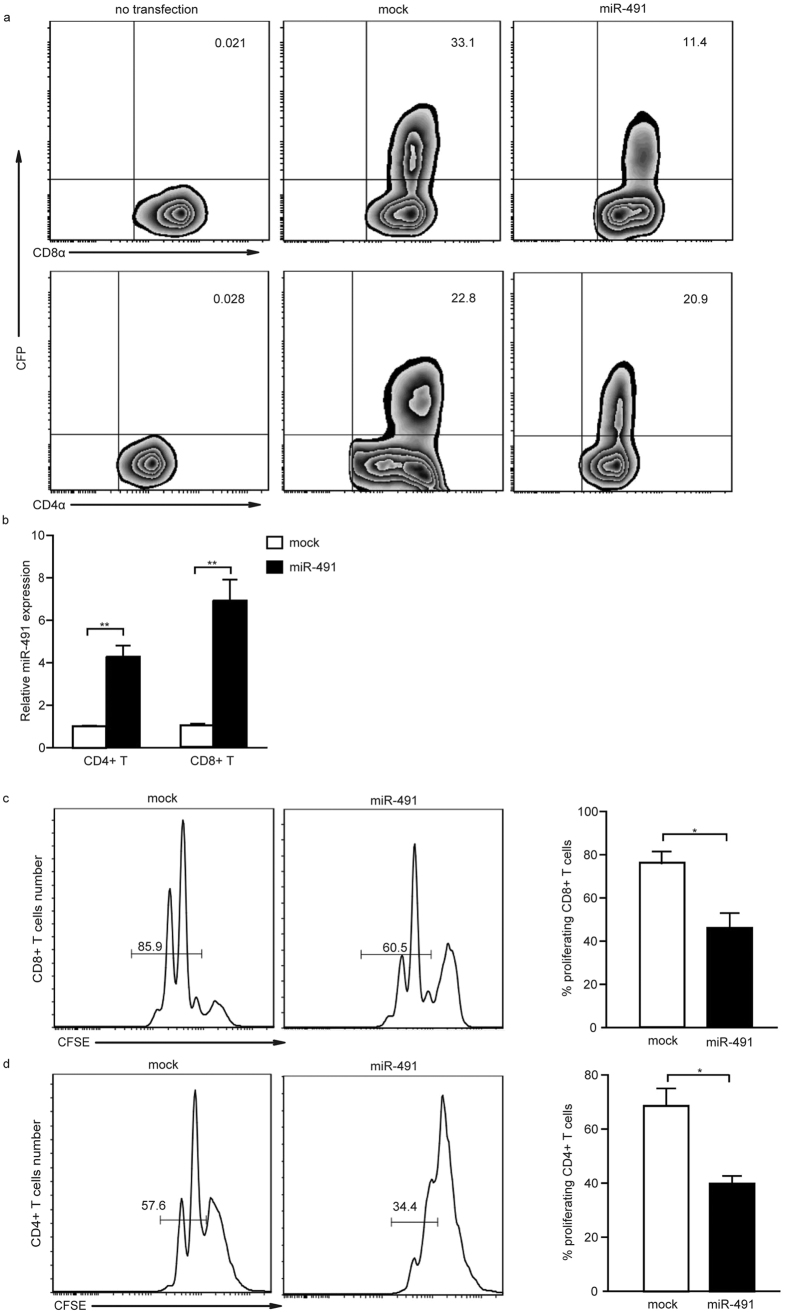
miR-491 inhibits T lymphocyte proliferation. (**a**) The transfection efficiency of a retroviral vector encoding the miR-491 transcript with CFP or CFP alone in CD8^+^ and CD4^+^ T cells. **(b)** After transfection with the retrovirus for 48 hours, miR-491 expression in CD8^+^ T cells or CD4^+^ T cells was separately tested by qRT-PCR. The bar graph shows the mean ± SD from three independent experiments. **P < 0.01. **(c,d)** Proliferation of miR-491-overexpressing T cells was detected by flow cytometry. The bar graph shows the mean ± SD from three independent experiments. *P < 0.05.

To characterize the role of elevated miR-491 in T cells, we constructed a retroviral vector to overexpress miR-491 in T cells. This process resulted in approximately 7- and 5-fold increases in miR-491 expression in CD8^+^ and CD4^+^ T cells, respectively (Fig. 2a,b). The overexpression of miR-491 limited T cell proliferation; the average proliferation rate was 45.4% *vs*. 76.03% in miR-491-overexpressing CD8^+^ T cells and mock cells (Fig. 2c), respectively, and 39.77% *vs*. 68.47% in miR-491-overexpressing CD4^+^ T cells and mock cells, respectively (Fig. 2d). To exclude the possibility that the observed phenotype caused by miR-491 was nonspecific, we overexpressed an irrelevant miRNA, miR-369, with the same retroviral expression vector and transduced CD8^+^ T cells. The results showed that miR-369 had no effect on proliferation of CD8^+^ T cells, and the role of miR-491 was not associated with the ectopic expression of vector sequences (Fig. S3). These results suggest that miR-491may impede the proliferation of both CD8^+^ and CD4^+^ T cells *in vitro*.

### miR-491 overexpression facilitated T cell apoptosis

**Figure 3:**
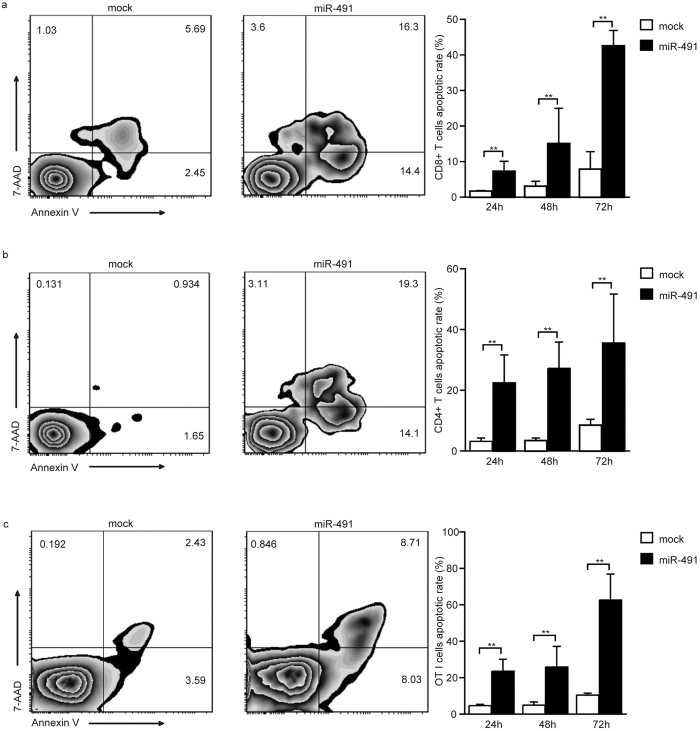
miR-491 promotes T cell apoptosis. (**a,b)** Apoptosis was induced in transfected CD8^+^ T cells and CD4^+^ T cells by treatment with anti-CD3 and anti-CD28 Abs for 24 hours, 48 hours and 72 hours. **(c)** Transfected OT-I splenocytes isolated from OT-I transgenic mice were induced to apoptosis by stimulation with an OVA-257 peptide. The apoptotic rates of these cells were assessed by annexin V/7-amino-actinomycin D (7AAD) staining. One representative flow cytometry analysis at 72 hours is shown. The bar graph shows the mean ± SD of three independent experiments. **P < 0.01.

After the immune response peaks, antigen-reactive T cells undergo apoptosis to maintain immune homeostasis. To determine whether miR-491 affects T cell survival, apoptosis was induced in miR-491-overexpressing T cells and mock cells by TCR stimulation. Then, apoptotic cells were analysed by flow cytometry. The results showed that miR-491 overexpression led to enhanced apoptosis in both CD8^+^ and CD4^+^ T cells (Fig. 3a,b). We further examined the role of miR-491 on OT-I cells. As shown in Fig. 3c, miR-491 overexpression also enhanced OT-I cell apoptosis. Overexpression of irrelevant miRNA, miR-369, was also performed and the results showed that miR-369 had no effect on T cells apoptosis (Fig. S4). These observations indicate that miR-491 is a positive regulator of T cell apoptosis induced by AICD *in vitro*.

In the tumour animal model, miR-491 was upregulated in spleen CD8^+^ T cells from tumour-bearing mice by ~2 fold. To mimic *in vivo* condition, we performed miR-491 overexpression by 2.2-fold in CD4^+^ T cells and 2.6-fold in CD8^+^ T cells. The results showed that even at about 2-fold upregulation, miR-491 could still limit CD4^+^ and CD8^+^ T cells proliferation and promote apoptosis (Fig. S5).

### miR-491 regulated IFN-γ production in CD8^+^ T cells

**Figure 4:**
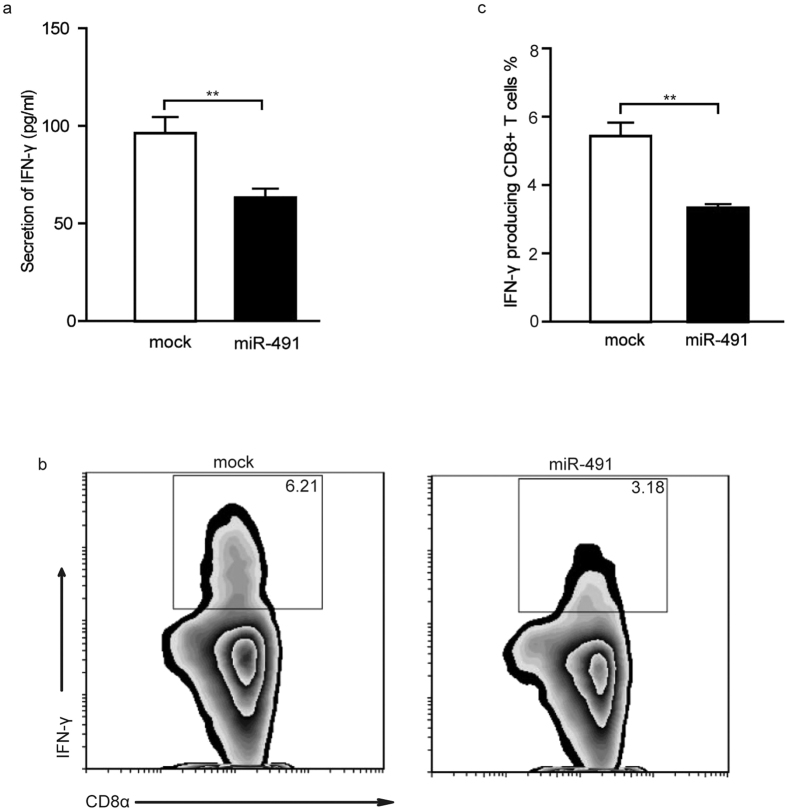
miR-491 regulates IFN-γ production. **(a**) The IFN-γ secreted by miR-491-overexpressing CD8^+^ T cells or mock cells was detected by ELISA. The reactions were repeated in six wells. **(b,c)** miR-491-overexpressing CD8^+^ T cells or mock cells were plated at 1 × 10^6^/ml in complete medium and stimulated with PMA (Sigma, 50 ng/ml), ionomycin (Sigma, 1 mM), and GolgiStop (BD Biosciences, 2 μl in 3 ml of culture medium) for 4 hours. The percentage of viable CD8^+^ T cells producing IFN-γ was determined by intracellular staining and flow cytometric analysis. The bar graph shows the mean ± SD of three independent experiments. **P < 0.01.

IFN-γ is a potent nonspecific tumour cell killer. To investigate whether miR-491 affects IFN-γ production, viable miR-491-overexpressing CD8^+^ T cells were selected and co-activated with anti-CD3 and anti-CD28 Abs for 4 days. Then, supernatants were collected to measure the levels of IFN-γ. We found that IFN-γ secretion was significantly decreased in miR-491-overexpressing CD8^+^ T cells compared with mock cells (Fig. 4a). To exclude the possibility that this decrease was caused by a defect in proliferation and survival, we examined cytokine production on a single-cell basis by intracellular staining. As shown in Fig. 4b,c, miR-491-overexpressing CD8^+^ T cells produced substantially less IFN-γ than mock cells. These observations suggest that miR-491 could dampen the antitumour ability of effector CD8^+^ T cells by inhibiting IFN-γ production.

### Bcl-xL, CDK4 and TCF-1 were direct targets of miR-491 in CD8^+^ T cells

**Figure 5:**
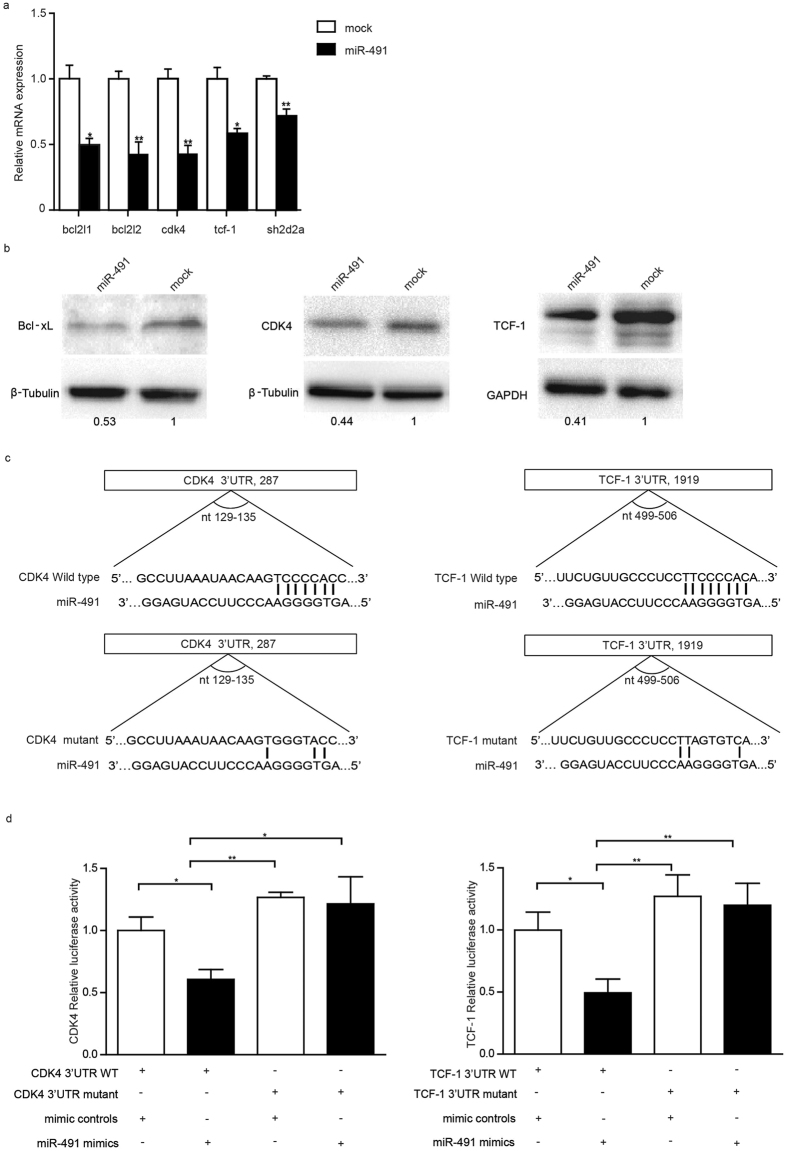
Prediction and validation of miR-491 target genes in CD8^+^ T cells. **(a)** RNA was extracted from miR-491-overexpressing CD8^+^ T cells and control cells, and qRT-PCR was performed as before. All PCR reactions were performed in triplicate. *P < 0.05; **P < 0.01. **(b)** Proteins extracted from miR-491-overexpressing CD8^+^ T cells and controls were analysed by western blotting. The data are normalized to the reference genes GAPDH or β-tubulin. **(c)** The graph shows the wild-type or mutant binding sites of CDK4-3′-UTR and TCF-1-3′-UTR for miR-491 and the construction model of the wild-type or mutant pLUC-CDK4-3′-UTR and pLUC-TCF-1-3′-UTR vectors. **(d)** Luciferase vectors containing wild-type or mutant CDK4-3′-UTR and TCF-1-3′-UTR were transfected with pRL-TK and miR-491 mimics or mimic controls. The luciferase activity was normalized to *Renilla* luciferase activity. The assays were repeated in five wells. *P < 0.05.

To investigate how miR-491 regulates the proliferation and apoptosis of CD8^+^ T cells, we searched for potential targets of miR-491 using bioinformatics tools (miRecords, http://mirecords.biolead.org/). *bcl2l1, bcl2l2, cdk4, tcf-1* and *sh2d2a* were chosen as candidate targets because these genes are involved in cell proliferation and survival. Overexpression of miR-491 in CD8^+^ T cells decreased the mRNA levels of *bcl2l1, bcl2l2, cdk4, tcf-1* and *sh2d2a* (Fig. 5a) but only decreased the protein levels of Bcl-xL (encoded by *bcl2l1*), CDK4, and TCF-1 (Fig. 5b). Bcl-xL has been shown to be a potent target of miR-491 in tumour cells^17^,^18^,^19^. We next performed luciferase assays to investigate whether CDK4 and TCF-1 were direct targets of miR-491. We separately constructed vectors containing wild-type or mutant miR-491 binding sites in the 3′-untranslated region (3′-UTR) of CDK4 mRNA or TCF-1 mRNA (Fig. 5c). The results showed that the relative luciferase activity was reduced by 40% in the vector containing the CDK4 3′-UTR and by 51% inthe vector containing the TCF-1 3′-UTR. However, miR-491 had no effect on mutant vectors (Fig. 5c). These results suggest that *tcf1*and *cdk4* are direct targets of miR-491.

### TGF-β1 induced miR-491 expression in CD8^+^ T cells

**Figure 6:**
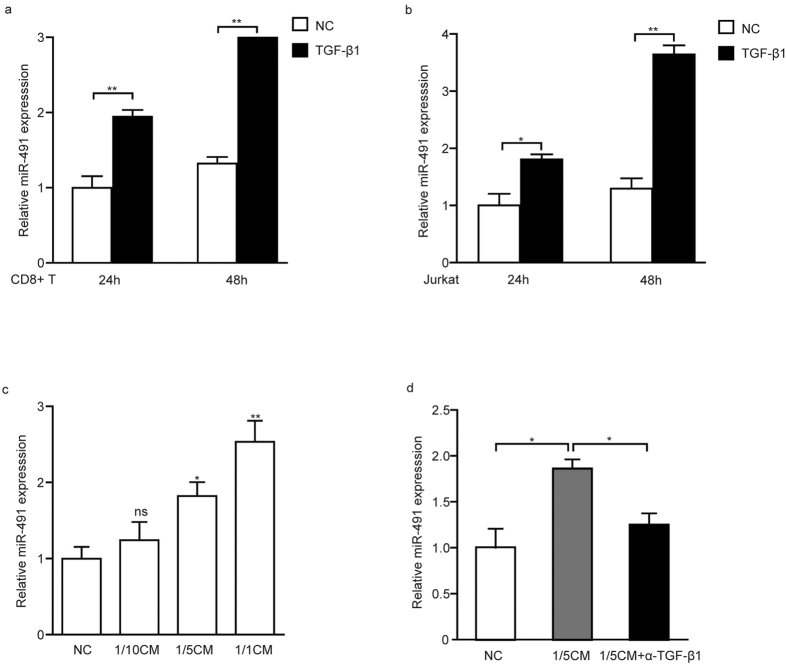
TGF-β1 upregulates miR-491 expression. Splenic CD8^+^ T cells **(a)** from C57BL/6 mice and Jurkat cells **(b)** were primed with anti-CD3 and anti-CD28 Abs for 24 hours and then treated with TGF-β1 (10 ng/ml) (R&D Systems, USA) for 24 and 48 hours. miR-491 expression was tested at the indicated time by qRT-PCR as described previously. Activated CD8^+^ T cells were co-cultured with fresh medium (negative control, NC) or serial dilutions of MC-38-CM **(c)** or MC-38-CM (1/5) plus anti-TGF-β1 (10 μg/ml) mAb for 24 hours **(d)**. Then, miR-491 expression was determined by qRT-PCR. All PCR reactions were performed in triplicate. *P < 0.05; **P < 0.01.

TGF-β plays an important role in regulating immune responses and homeostasis^21^. The elevated TGF-β level during tumour progression is often regarded as immunosuppressive. Notably, miR-491 has been reported to be induced by TGF-β1 in rat proximal tubular epithelial cells^22^. Thus, we next examined whether TGF-β1 could modulate miR-491 expression in CD8^+^ T cells. The results showed that TGF-β1 treatment led to a dramatic increase in miR-491 in mouse CD8^+^ T cells by 1.94- and 2.74-fold at 24 hours and 48 hours (Fig. 6a), respectively. In Jurkat cells, a human T cell line, TGF-β1 caused a 1.81- and 2.81-fold upregulation of miR-491 at 24 hours and 48 hours (Fig. 6b), respectively. To explore whether miR-491 expression was also regulated by tumour cell-derived TGF-β, activated CD8^+^ T cells were co-cultured with diluted MC-38 colorectal cancer cell-conditioned medium (MC-38-CM) for 24 hours. As expected, miR-491 expression was significantly upregulated (Fig. 6c). Moreover, the induction of miR-491 in CD8^+^ T cells was partially blocked by an anti-TGF-β1 mAb co-cultured with MC-38-CM (Fig. 6d). These data suggest that miR-491 is regulated by tumour-derived TGF-β in CD8^+^ T cells in a time-dependent manner.

## Discussion

**Figure 7:**
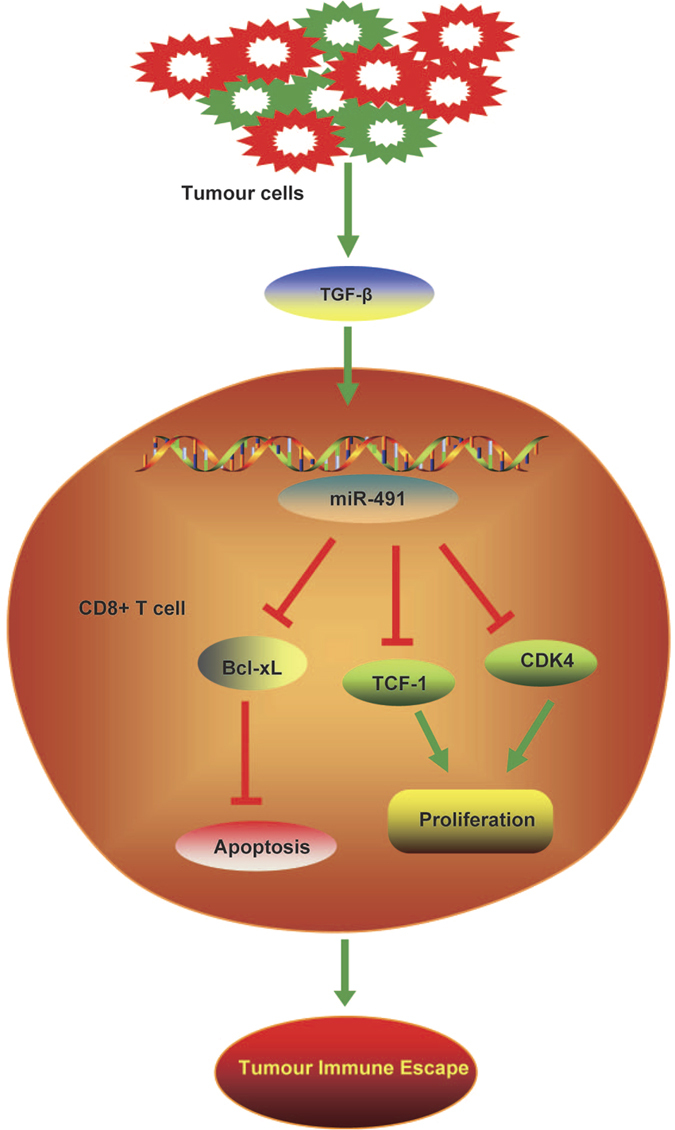
Model illustrating the role of miR-491 in CD8^+^ T cells. Tumour-derived TGF-β induces upregulation of miR-491 in CD8^+^ T cells. miR-491 inhibits activated CD8^+^ T cell proliferation and promotes apoptosis by targeting Bcl-xL, CDK4 and TCF1, which results in tumour immune escape. The figure was drawn by ScienceSlides 2005 edition.

CTLs are required to fight tumourigenesis and cancer progression. Several reports have shown that the enhanced proliferation and function of CTLs can prolong the disease-free survival of tumour patients^23^,^24^. In this study, we determined that tumour-derived TGF-β could induce the expression of miR-491 in CD8^+^ T cells from colorectal tumour-bearing mice compared with their non-malignant counterparts. miR-491 overexpression in T cells may compromise the antitumour ability by limiting cell proliferation, promoting cell apoptosis and decreasing IFN-γ production in CD8^+^ T cells. These functions may be induced by inhibition of Bcl-xL, CDK4 and TCF-1, which are involved in the proliferation, survival and function of T cells. Overall, miR-491 acted as a negative regulator of T cells, especially CD8^+^ T cells, in the tumour environment and may favour tumour immune escape (Fig. 7).

CDK4 is an important cell cycle regulator, and the inhibition of CDK4 results in the decreased proliferation of tumour-specific effector T cells^25^. TCF-1 is a transcriptional activator that plays an important role in lymphocyte differentiation. Decreased expression of TCF-1 limits the proliferation of effector CD8^+^ T cells, reduces the expression of the anti-apoptotic molecule Bcl-2, and impairs the establishment of functional memory CD8^+^ T cells^26^,^27^,^28^. Therefore, miR-491, which is induced in colorectal tumour-bearing mice, may negatively affect the proliferation of CD8^+^ T cells by targeting CDK4 and TCF-1. Bcl-xL, another identified target of miR-491 in our study, is an anti-apoptotic molecule that belongs to the Bcl-2 family. It is well known that activated T cells could die via intrinsic pathway. The intrinsic pathway is triggered by proteins that regulate cell death, such as the Bcl-2 family proteins, particularly Bcl-2, Bcl-xL, Bim, and probably Bak^29^. The enforced expression of Bcl-xL in Jurkat cells almost completely rescued cells from apoptosis^30^. Therefore, the increased sensitivity of miR-491-overexpressing T cells to AICD may be attributed to the inhibition of the anti-apoptotic molecule Bcl-xL. We also discovered that miR-491-overexpressing CD8^+^ T cells produced less IFN-γ compared with mock cells. IFN-γ collaborates with lymphocytes to protect against the development of cancer^31^. This result indicated that miR-491 could compromise the antitumour ability of CD8^+^ T cells partially through the inhibition of IFN-γ.

Furthermore, we found that miR-491 expression was upregulated by TGF-β1, which is known to limit the activation and function of CD8^+^ T cells. TGF-β suppresses the effector functions of CTLs in multiple ways, such as by inhibiting IFN-γ expression or reducing the clonal expansion of CD8^+^ T cells^21^. TGF-β can also promote T cell death by regulating the expression of the Bcl-2 family during infectious diseases^32^,^33^. It has been reported that tumour-derived TGF-β has critical roles in inhibiting host immunosurveillance^34^. Except for miR-491, TGF-β can alter the expression of numerous miRNAs in CD8^+^ T cells^35^. In human CD8^+^ T cells, miR-23a cluster (miR-23a, miR-27a, miR-24), and miR-720 were upregulated by TGF-β^36^,^37^. In mouse CD8^+^ T cells, miR-23a was effectively upregulated by TGF-β in TCR-activated CTLs and mediated tumor immune evasion^38^. Our results indicated that TGF-β produced by tumour cells could modulate miR-491 expression and hence negatively regulate the proliferation and survival of T cells, but it needs further investigation to enclose the mechanism. These results suggest that TGF-β may contribute to systemic immune suppression and facilitate tumour immune escape in a miRNA-dependent manner.

To overcome tumour-induced T cell dysfunction, many effective immunotherapies have been developed and have become important strategies for the treatment of tumours. However, there are still some constraints. For example, monoclonal antibodies targeting immune checkpoints, such as CTLA-4 and PD1, can indeed result in a sustained antitumour response by broadening the antigen-reactive CD8^+^ T cell response^39^,^40^, but they may cause serious toxicity. Moreover, anti-inhibitory factor therapy can also activate autoimmunity, and therefore, these therapies should not be used by patients with autoimmune diseases^41^. Anticancer vaccines usually cannot effectively eliminate tumours because persistent antigens convert tumour-specific effector CD8^+^ T cells into dysfunctional, even apoptotic, cells, thus leading to an ineffective immune response and hyporesponsiveness to subsequent vaccination^42^. During adoptive cell transfer (ACT) therapy, the toxicities induced by high-intensity preconditioning regimens and high-dose IL-2 can be severe and sometimes lethal^43^. These limitations have prompted investigators to combine classical immunotherapies with other immunomodulatory targets, such as miRNAs, which closely correlate with the development and function of the immune system. For example, miR-155 can be used to increase the effectiveness of ACT therapy in a cell-intrinsic manner in the absence of lymphodepletion preconditioning and cytokine administration^44^. Moreover, miR-155 expression has been associated with the chemoimmunotherapy outcome of patients with chronic lymphocytic leukaemia and can be used as a biomarker to classify patients receiving chemoimmunotherapy^45^. The inhibition of miR-23a, an inhibitor of CTL cytotoxicity, has been shown to preserve immune competence in the tumour microenvironment and enhance ACT therapy^46^. miRNAs can also be used in combination with monoclonal antibodies targeting inhibitor molecules. For example, it has been reported that miR-29 can downregulate the expression of B7-H3, which functions as an inhibitor of natural killer cells and T cells. Combined treatment with miR-29a and 8H9, a specific mAb to B7-H3, may advance both cell-mediated immunotherapy and antibody-based targeted strategies^47^. These studies provide evidence that antitumour immunotherapy can be optimized via the modulation of miRNAs.

miRNAs are important regulators of CD8^+^ T cell differentiation, activation, migration and function. miR-150 and miR-17–92 cluster are required for robust effector CD8^+^ T cell proliferation and functional memory CD8^+^ T cell formation^48^,^49^,^50^,^51^. miR-17–92 cluster promoted CD8^+^ T cell migration to graft-versus-host disease (GVHD) target organs^52^. miR-130/301 impeded CD8^+^ T cells migration out of the central lymphoid organs after T cell activation^53^. miR-139, miR-342 and miR-150 have been shown to form a miRNA network that suppresses perforin, eomesodermin, and CD25 expression in differentiating CTLs^54^. In tumour microenvironment, miR-101 and miR-26a decreased the frequency of polyfunctional CD8^+^ T cells and enhanced CD8^+^ T cell apoptosis thus favouring tumour immune evasion^55^. In tumour-infiltrating CTLs, miR-23a repressed the transcription factor BLIMP-1 and led to impairment of effector cell differentiation and CTLs cytotoxicity^38^.

It is well known that miRNAs expressed at relatively high levels are more likely to have biological functions. However there are still some evidence to show that miRNAs with moderate or relatively low level may have biological roles or prognostic roles, such as miR-31, miR-214, miR-125 b and miR-138^56^,^57^,^58^,^59^. For instance, miR-31 is not highly expressed in human PBMCs, but it functions as regulator of T cell survival and can be used as a biomarker to predict survival of patients with HIV^56^. miR-214, whose expression level is comparable to miR-491 in mouse CD8^+^ T cells, is significantly upregulated upon T cell activation and could promote T cells proliferation by targeting *pten*^57^. miR-491 has been reported to be existed in astrocyte and several kind of epithelial cells before^16^,^17^,^18^. In our study, miR-491 was indeed not abundant in CD8^+^ T cells. But it was upregulated in CD8^+^ T cells, especially in effector-like CD8^+^ T cells, from tumour-bearing mice than controls. Furthermore, miR-491 could apparently regulate T cell proliferation and apoptosis and IFN-γ production. This evidence suggests that miR-491 may have physiological significance CD8^+^ T cells *in vitro* even at a relative low level.

miR-491 acts as a tumour suppressor in several tumours. miR-491 could effectively induce tumor cell apoptosis and inhibit cell proliferation by targeting Bcl-xL and EGFR^17^,^18^,^19^. miR-491 impaired focal adhesion and EGFR/ERK1/2 signalling pathways through targeting GIT1 and MMP9, then suppressed tumour invasion and metastasis^16^,^60^. Lower expression of miR-491 correlated with poor overall survival of patients with oral squamous cell carcinoma^16^. In breast cancer, miR-491 was identified as an inhibitor of HER2 signalling and induced tumour cell apoptosis^20^. To our knowledge, this is the first report to study the role of miR-491 in T cells. However, additional investigations are needed to determine the effect of miR-491 in CD8^+^ T cell subsets during tumour development *in vivo*.

Notably, in the present study, miR-491 is identified as a negative regulator of T cell function and may contribute to tumour immune evasion. This distinctive conclusion compared with that in tumour cells is consistent with the consensus that the function of miRNAs is context and cell specific^61^. Taking together these findings, we show that miR-491 may have a double-edged role in the tumour environment and suggest that alternative manipulation of miR-491 may be required in different cell types during tumour progression. On one hand, the inhibition of miR-491 expression in CTLs may promote their cytotoxic ability and restrict tumour progression. On the other hand, it is necessary to induce miR-491 expression in some tumour cells to perform their antitumour function. These different treatments will be important to optimize the antitumour immune response.

## Materials and Methods

### Ethics statement

All animal care and use protocols in this study were performed in accordance with the Regulations for the Administration of Affairs Concerning Experimental Animals approved by the State Council of People’s Republic of China. All animal experiments were approved by the Animal Ethical and Experimental Committee of the Third Military Medical University (Chongqing, China).

### Animal model

Female C57BL/6 mice 6–8 weeks old of age were purchased from Beijing HFK Bioscience Limited Company (Beijing, China) and maintained under specific pathogen-free conditions. MC-38 colorectal cancer cells (1 × 10^6^) were suspended in 100 μl of phosphate-buffered saline (PBS) and injected subcutaneously into the right lateral flank of the mice, and the control mice were injected subcutaneously with 100μl PBS into the right lateral flank (five mice per group). Thirty days later, the tumour volumes were approximately 1 × 1 cm (length × width). The mice were sacrificed by cervical dislocation.

### Cell lines and T cell activation

The MC-38 colorectal cancer cell line was kindly provided by Professor Li-Guo Zhang (Chinese Academy of Sciences, Beijing, China). MC-38 cells, EL4 cells, BOSC 23 cells and HEK-293 cells were cultured in DMEM plus 10% FBS at 37 °C and 5% CO_2_. For the collection of MC-38-CM, subconfluent MC-38 cells were kept in serum-free DMEM for 24 hours. Then, the supernatants were collected, aliquoted, and stored at −80 °C until use. Jurkat cells were cultured in RPMI 1640 plus 10% FBS under standard conditions.

Spleen- or lymph node-derived T cells were isolated from C57BL/6 mice. CD8^+^ T cell were enriched using positive selection beads (Miltenyi Biotec, Auburn, CA, USA). Flow cytometry analysis showed that CD8^+^ T cells were >95% pure. T cells were activated by plate-bound anti-CD3 (2 μg/ml, BioLegend) antibody plus soluble anti-CD28 (5 μg/ml, BioLegend) antibody in RPMI 1640 plus 10% FBS and 50 U/ml recombinant human (rh) IL-2 (PeproTech, USA) for 24 hours. OT-I splenocytes were derived from OT-I transgenic mice (Jackson Laboratory) and stimulated with the OVA-257 peptide SIINFEKL (1 μmol/L) overnight.

### Retroviral transduction

BOSC 23 cells were co-transfected with retroviral expression plasmids, either encoding a miR-491 transcript and CFP or CFP alone (mock), and pCL-Eco retrovirus packaging vector using Lipofectamine LTX and PLUS (Invitrogen). Supernatants were collected 48 hours later and stored at −80 °C for use. Activated T cells were plated in a 24-well plate and spin-infected with retrovirus at 1258 rcf and 37 °C for 90 minutes. Then, the supernatants were replaced by fresh complete RPMI 1640 plus 10% FBS and 50 U/ml rh IL-2. The cells were continually cultured for another 48 hours under standard conditions.

### RNA extraction and qRT- PCR

Total RNA was extracted from splenic CD8^+^ T cells from tumour-bearing or healthy mice with TRIzol (Invitrogen) and then used for a qPCR-based high-throughput miRNA array containing primers specific for 345 well-characterized miRNAs. Retrovirus-transfected CD8^+^ T cells were isolated by flow cytometry, and RNA was extracted by a mirVana miRNA isolation kit (Life Technologies, Carlsbad, CA, USA). The expression of miRNAs was analysed by a TaqMan microRNA assay kit (Applied Biosystems), and U6 snRNA was used as a reference gene. Reverse transcription of mRNA was performed with a PrimeScript RT Reagent Kit (TaKaRa), and the qPCR was performed with SYBR Green Real Time PCR Master Mix (TOYOBO). Gapdh was used as a reference gene. The primers used in this study are shown in Table. S1. The Bio-Rad CFX96 real-time PCR system (Bio-Rad, Hercules, CA, USA) was used. All qRT-PCR reactions were performed in triplicate, and the data were analysed with Bio-Rad CFX Manager software version 3.1.

### Cell proliferation and apoptosis assays

Cell proliferation and apoptosis rates were determined by flow cytometric analysis. For the analysis of proliferation, miR-491-overexpressing T cells and mock cells were isolated by density gradient centrifugation and labelled with carboxyfluorescein diacetate succinimidyl diester (CFSE) (5 μM, eBioscience) for 10 minutes at room temperature, and the reaction was stopped with one volume of foetal calf serum. Then, the cells were washed three times with PBS. These cells were continually cultured for another 48 hours under standard conditions, and the proliferation rate was assessed. For the apoptosis experiment, miR-491-overexpressing T cells and mock cells were co-activated by anti-CD3 and anti-CD28 Abs for the indicated time. OT-I T cells after transfection were isolated and restimulated with the OVA-257 peptide SIINFEKL for the indicated time. Then, cells were double-stained by annexin V/7AAD (BioLegend), and the rate of apoptosis was assessed. The results were analysed by FlowJo software version 6.2.

### Enzyme-linked immunosorbent assay (ELISA)

After retrovirus transfection, miR-491-overexpressing CD8^+^ T cells and mock cells were isolated by flow cytometry. Then, these cells were continually cultured for 4 days in complete RPMI 1640 with 10% FBS, anti-CD3 (2 μg/ml) and anti-CD28 (5 μg/ml) Abs and rhIL-2 (50 U/ml). The supernatants were then collected to detect IFN-γ levels by ELISA (Abcam). A SpectraMax 190 microplate reader (Molecular Devices) was used.

### Western blot assay

Proteins were collected from miR-491-overexpressing CD8^+^ T cells or mock cells with RIPA buffer (Pierce). Protein concentration was detected by bicinchoninic acid protein assays (Pierce). Samples (10 μg/lane) were separated by 10% SDS-polyacrylamide gel electrophoresis and transferred to polyvinylidene fluoride membranes (Millipore). The primary anti-GAPDH (1:5000) and anti-β-tubulin (1:5000) mouse monoclonal antibodies and anti-TCF-1 (1:1000) and anti-Bcl-xL (1:1000) rabbit monoclonal antibodies were purchased from Cell Signaling Technology. The anti-CDK4 (1:1000) monoclonal antibody and the HRP-conjugated secondary antibody (1:10000) were purchased from Abcam. GAPDH and β-tubulin served as internal references. Bound proteins were visualized by using the SuperSignal West Dura Extended Duration Substrate Kit (Thermo Scientific, China). The data were analysed using ImageJ.

### Luciferase assay

The whole 3′-UTR of CDK4 and TCF-1 were amplified from genomic DNA of the EL4 cells by PCR. Mutational 3′-UTRs were constructed by replacing 4 or 5 nucleotides in the miR-491 binding sites with PCR repair. The primers were listed in Table. S2. Then, the wild-type 3′-UTRs and mutational 3′-UTRs were individually cloned downstream of the firefly luciferase gene in the pMIR-REPORT luciferase miRNA expression reporter vector (Ambion). HEK293 cells were seeded in a 96-well plate (1 × 10^4^ per well), and each well was co-transfected with 0.2 μg of wild-type or mutant firefly luciferase reporter vectors, 0.01 μg of pRL-TK (Promega) and miRNA-491 mimics (50 nM) or negative-control scrambled miRNA mimics (50 nM) (RiboBio, China) using Lipofectamine 2000 (Invitrogen). Twenty-four hours later, the cells were lysed, and fluorescence activity was detected via dual luciferase reporter assay system (Promega). The firefly luciferase activity was normalized to *Renilla* luciferase activity.

### Statistical analysis

Student’s t tests were used to analyse the differences between two groups. A *P* value < 0.05 was considered significant. The data were analysed with GraphPad Prism software version 5.0.

## Additional Information

**How to cite this article**: Yu, T. *et al*. MicroRNA-491 regulates the proliferation and apoptosis of CD8^+^ T cells. *Sci. Rep*. **6**, 30923; doi: 10.1038/srep30923 (2016).

## Supplementary Material

Supplementary Information
