# Supplementary material for: MicroRNA-491 regulates the proliferation and apoptosis of CD8+ T cells
Source: Sci Rep. 2016 Aug 3;6:30923. doi: 10.1038/srep30923 (PMC4971478; doi:10.1038/srep30923)
Supplement: Supplementary Information [file srep30923-s1.doc]

**MicroRNA-491 regulates the proliferation and apoptosis of CD8^+^ T cells**

Ting Yu^1^, Qian-Fei Zuo^1^, Li Gong^1^, Li-Na Wang^1^, Quan-Ming Zou^1*^ and Bin Xiao^1*^

**Supplementary Figures**


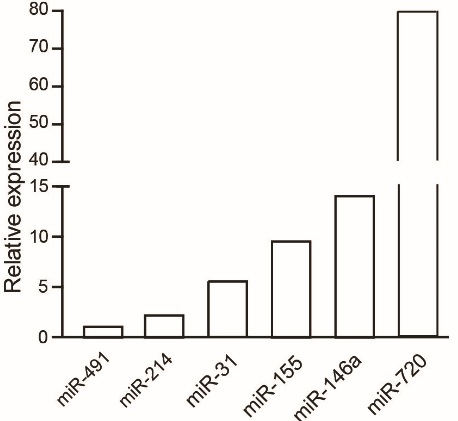


**Figure S1 Expression of miR-491 and other more abundant miRNAs in CD8^+^ T cells.** Freshly splenic CD8^+^ T cells were isolated from mice and RNA was extracted, then the expression of several selected miRNAs was validated by qPCR. U6 was used as an internal reference.


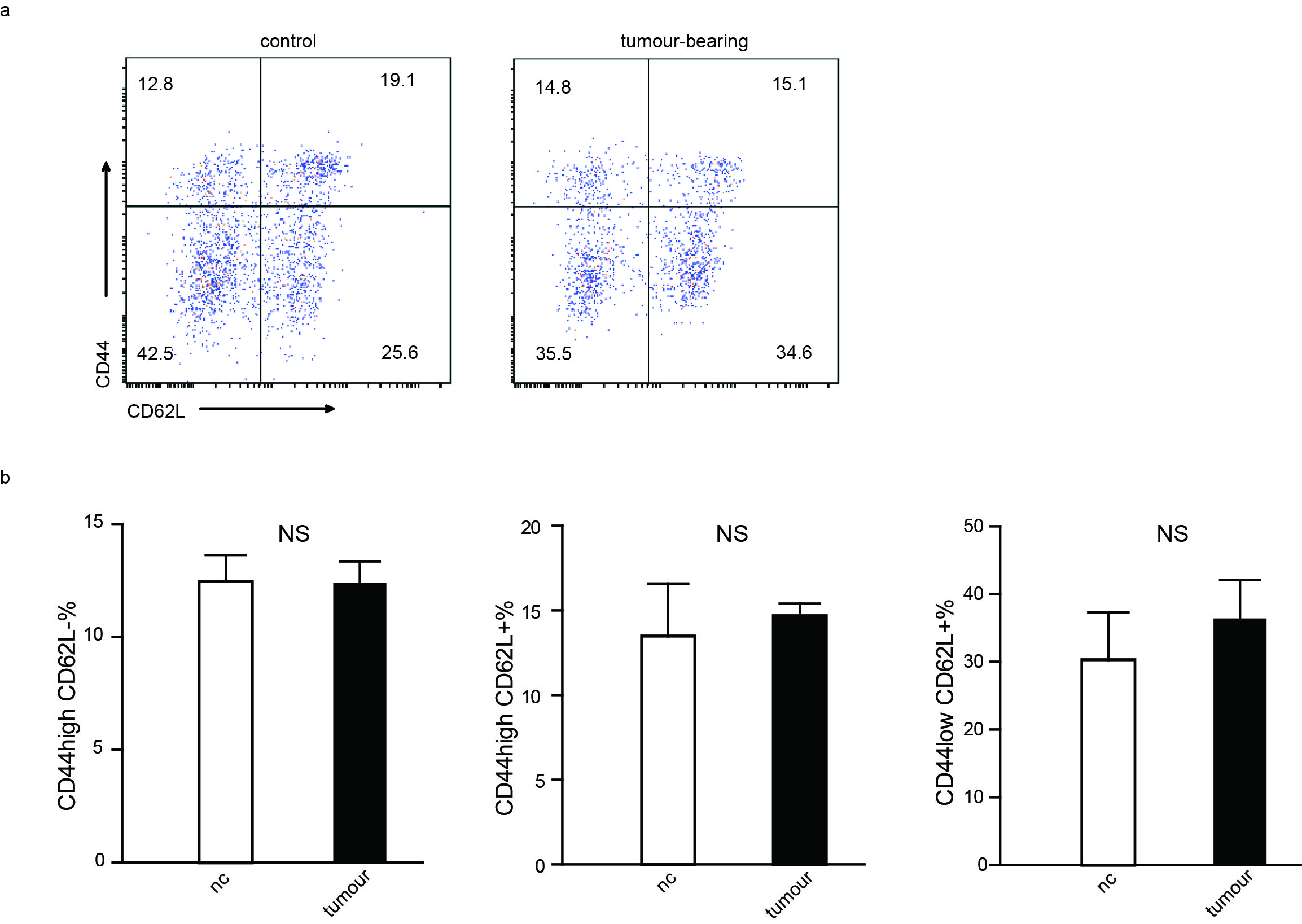


**Figure S2 Percentages of CD8^+^ T cell subsets between tumour-bearing group and controls**. (a) Flow cytometric analysis of CD8^+^ T cell subsets in healthy controls and tumour-bearing mice. (b) Percentages of effector-like, memory, and naïve CD8^+^ T cells between healthy controls and tumour-bearing mice. (n=5 *vs* 5).


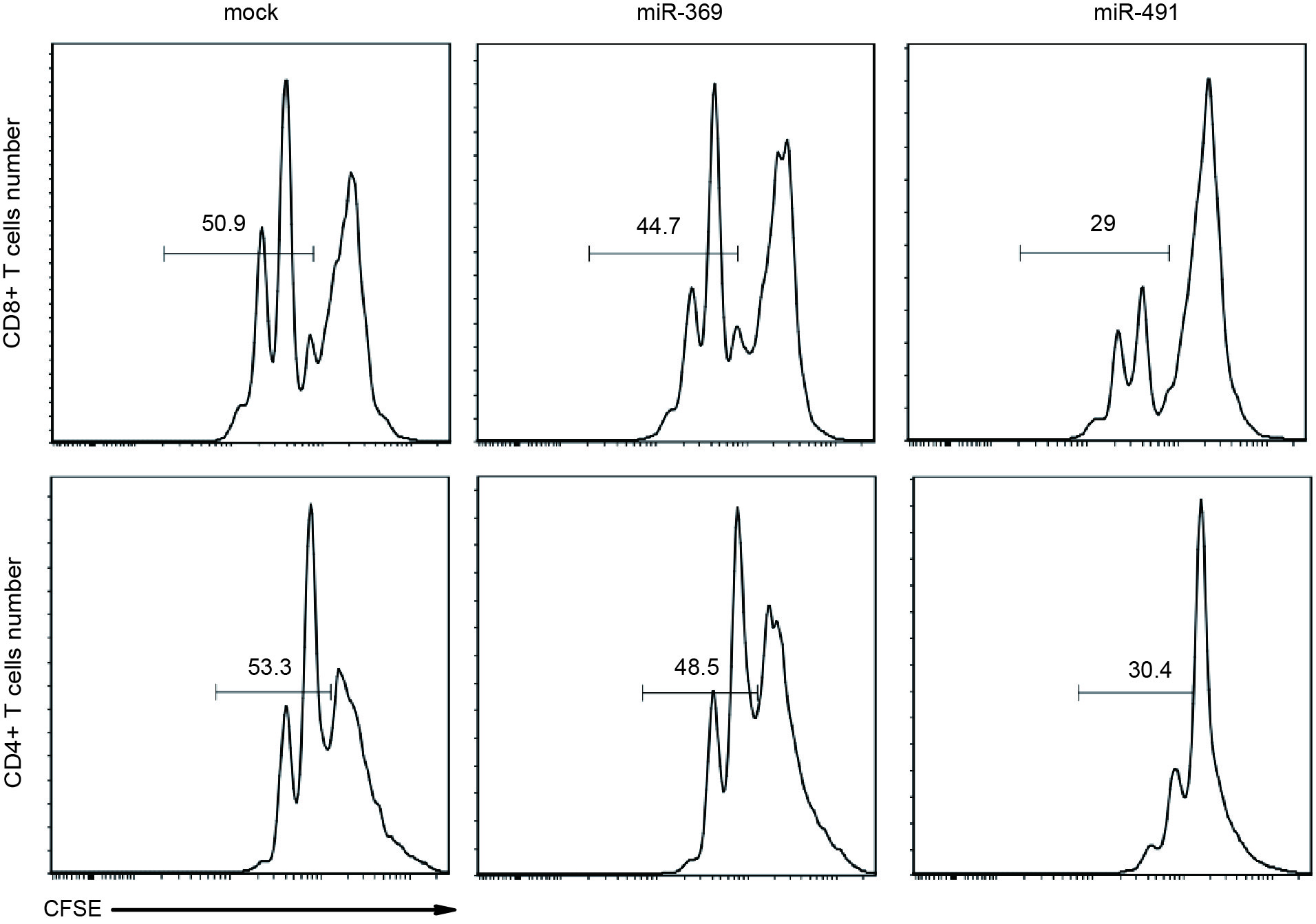


**Figure S3 Effect of miR-369, miR-491 and empty vector on T cell proliferation.** T cells were primed with anti-CD3, anti-CD28 antibodies for 24h, then cells were transduced with miR-369-expressing retrovirus, miR-491-expressing retrovirus, and empty retrovirus for 48h, and then stained with CFSE. The proliferation rates were detected 48 hours later.


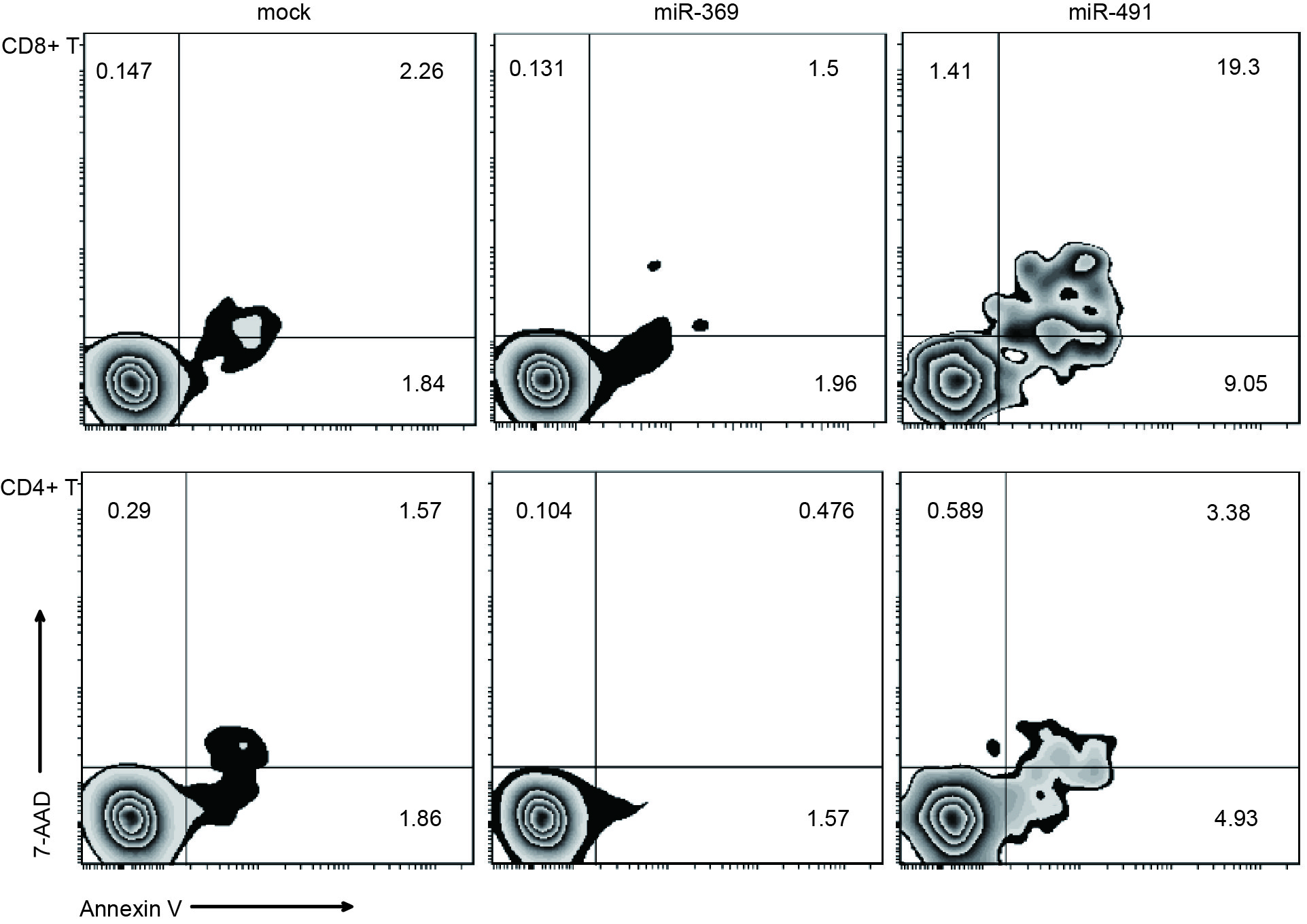


**Figure S4 Effect of miR-369, miR-491 and empty vectors on T cell apoptosis.** T cells were primed with anti-CD3, anti-CD28 antibodies for 24h, then cells were transduced with miR-369-expressing retrovirus, miR-491 expressing retrovirus, and empty retrovirus for 48h, and then the apoptosis rates were detected with Annexin V/7-AAD staining 72 hours later.


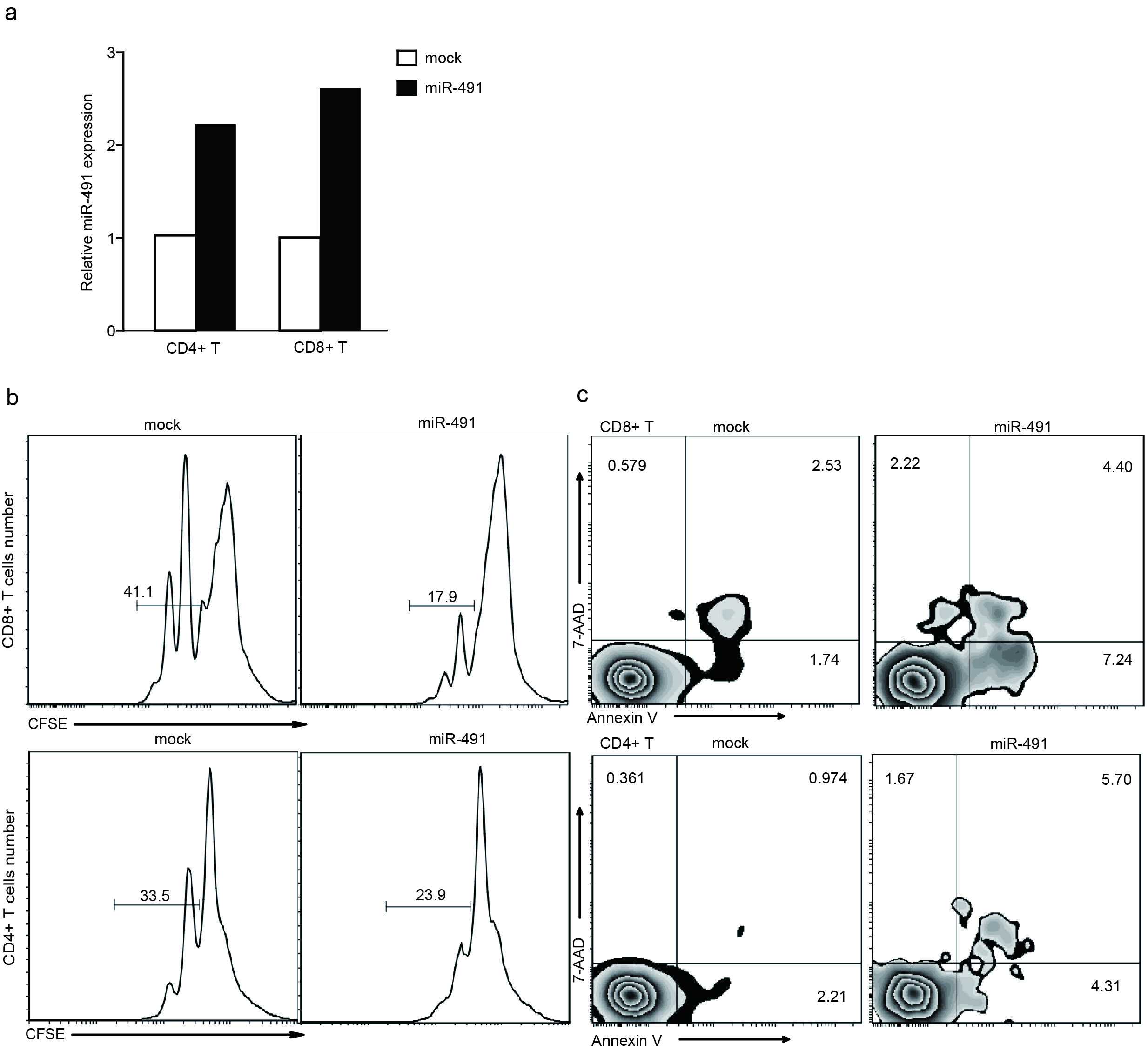


**Fig. S5 miR-491 upregulated by about 2-fold can also inhibits T lymphocyte proliferation and promotes T lymphocyte apoptosis. (a)** After transfection with retrovirus for 48 hours, CD4^+^CFP^+^ cells and CD8^+^CFP^+^ cells were sorted by FACS and expression of miR-491was tested by qRT-PCR. **(b)** Transfected T cells were stained with CFSE and cultured for 48 hours. Then proliferation of T cells was detected by flow cytometry. **(c)** Transfected T cells were induced to apoptosis by challenged with anti-CD3 and anti-CD28 Abs for 48 hours. Then apoptotic cells were assessed by annexin V/7AAD staining.

**Table.S1 Quantitative realtime PCR primer sequences**

| **Primers** | **Sequence** |
| --- | --- |
| gapdh-F | AGGTCGGTGTGAACGGATTTG |
| gapdh-R | GGGGTCGTTGATGGCAACA |
| bcl2l1-F | CCGGTCTCTTCAGGGGAAAC |
| bcl2l1-R | CCCGGTTGCTCTGAGACATT |
| bcl2l2-F | TAGAGTACCTGCCATGACC |
| bcl2l2-R | AGCATTAAAGAGCAGCAAT |
| cdk4-F | AATGTTGTACGGCTGATGGA |
| cdk4-R | AGAAACTGACGCATTAGATCCT |
| tcf-1-F | AGAAGCAAGGAGTTCACAGG |
| tcf-1-R | TGTCTATATCCGCAGGAAGGG |
| sh2d2a-F | AACATTACACAGAGTGCCC |
| sh2d2a-R | TCCTGTCTTTTGCTTCCAGAG |

Table.S2 The cloning primers of microRNA target region

| **Primers** | **sequences** |
| --- | --- |
| cdk4 3'UTR-F | AGTGAGCTCGAAGAGGGGCTGCCTTTCCCAGTCTTGG |
| cdk4 3'UTR-R | ACTACGCGTGTCTTGTCTTGTTTTCCTGTATAAAA |
| cdk4 mut-P1 | CTCGTAAGGAGAGATAAAAACTTGTTATTTAAGGCTTAA |
| cdkf mut-P2 | TATCTCTCCTTACGAGGTTCACCCCCATTACCCTCCCCT |
| cdk4 mut-P3 | CTCGTAAGGAGAGGTGGGGACTTGTTATTTAAGGCTTAA |
| cdkf mut-P4 | CACCTCTCCTTACGAGGTTCACCCCCATTACCCTCCCCT |
| tcf1 3'UTR-F | AGTGAGCTCGCTGTCCCCGGTCCCC |
| tcf1 3'UTR-R | ACTACGCGTGACTTTGAAAAACCAAGTAAAA |
| tcf1 mut-P1 | CTCACCCTCGTATCTTCTGTTGCCCTCCTATTTTATAGA |
| tcf1 mut-P2 | TCCACTGGGCTAGCAAGCAGTTCTATAAAATAGGAGGGC |
| tcf1 mut-P3 | CTCACCCTCGTATCTTCTGTTGCCCTCCTTCCCCACAGA |
| tcf1 mut-P4 | TCCACTGGGCTAGCAAGCAGTTCTGTGGGGAAGGAGGGC |
